# Leakage sign for acute subdural hematoma in clinical treatment

**DOI:** 10.1007/s00701-018-3755-x

**Published:** 2018-12-17

**Authors:** Masafumi Yamamoto, Kimihiko Orito, Yukihiko Nakamura, Nobuyuki Takeshige, Munetake Yoshitomi, Yasuharu Takeuchi, Hideaki Uzu, Osamu Takasu, Toshi Abe, Shuichi Tanoue, Yuusuke Uchiyama, Motohiro Morioka

**Affiliations:** 1 Department of Neurosurgery, Kurume University School of Medicine, 67 Asahimachi, Kurume, 830-0011 Japan; 2 Department of Emergency and Acute Intensive Care Medicine, Kurume University School of Medicine, Fukuoka, 830-0011 Japan; 3 Department of Radiology, Kurume University School of Medicine, Fukuoka, 830-0011 Japan

**Keywords:** Hematoma expansion, Leakage sign, Subdural hematoma, Computed tomography angiography

## Abstract

**Background:**

Acute subdural hematoma (ASDH) is a serious traumatic disease, and predictive methods for hematoma growth are necessary to decide whether emergent operation is necessary. This study aimed to evaluate the incidence of “leakage” using computed tomography angiography (CTA) in patients with ASDH and to identify its prognostic value.

**Methods:**

Sixty-seven patients with ASDH were examined using CTA (mean age 64.1 ± 20.6 years; 24 men) by analyzing two serial scans (CTA phase and delayed phase). We defined a positive leakage sign as a > 10% increase in Hounsfield units (HU) in the region of interest. Hematoma expansion was determined using plain CT after 24 h in patients who did not undergo emergent surgery.

**Results:**

Of the 67 patients, conservative therapy was administered to 35 patients; of these patients, 9 showed hematoma expansion, and 8 of these 9 patients (88.9%) showed positive leakage signs. The sensitivity and specificity of leakage signs to hematoma expansion in the no-surgery group were 88.8% and 76.1%, respectively. All positive leakage signs were found within 4.5 h of injury; patients showing negative leakage signs showed a decreased tendency towards hematoma 24 h after injury. Patients presenting with positive leakage signs had poor outcomes.

**Conclusions:**

The results indicated that the leakage sign is a sensitive predictor of hematoma expansion and poor outcomes in ASDH. If the hematoma is small but leakage sign-positive, strict observation is necessary and aggressive surgery may improve outcomes.

## Introduction

Acute subdural hematoma (ASDH) is a serious disease with high morbidity and mortality. Many cases require emergent operation on admission to prevent brain herniation. Contrarily, some patients with a small hematoma and faint disturbance of consciousness on admission show a delayed, sudden increase in hematoma size, whereas other cases show no increase in hematoma size, resulting in good outcomes. Thus, the timing and decision of surgical intervention is an important issue for ASDH patients [5, 6, 14]. Correctly predicting the expansion of the hematoma is crucial. This prediction helps in choosing aggressive surgery and avoids unnecessary surgical operations.

We have previously reported a sensitive predictive method named “leakage sign” for contusional hematoma cases, with high sensitivity, specificity, and predictive value for hematoma expansion [9]. The purpose of this study was to establish a sensitive predictive method for ASDH expansion using this leakage sign. We expected that the leakage sign would be valuable in the selection of optimal operative strategy.

## Materials and methods

### Patient selection

All patients with traumatic head injury that were transferred to our institute between April 2012 and August 2015 were initially included in this prospective study (*n* = 152). We performed computed tomography angiography (CTA) on all patients with ASDH to determine whether any vascular lesions were present. If for any reason CTA could not be performed, the patient was excluded. Patients with chronic subdural hematoma, patients with Glasgow Coma Scale (GCS) score of 3 points with bilateral dilated pupils, patients allergic to the contrast medium, patients with kidney dysfunction, and patients with only diffuse axonal injury or traumatic subarachnoid hemorrhage were excluded. CTA was not performed for patients with rapidly progressive symptoms, and they were also excluded. A total of 67 cases of ASDH were included in this prospective study. This study was approved by the review board and Ethics Committee of our institution. Informed consent was obtained from all patients.

### Clinical data

The following patient clinical data were recorded at admission: age, sex, arterial blood pressure, and the time from onset to admission. In addition, coagulation status at admission was evaluated using the international normalized ratio, prothrombin time, partial thromboplastin time, and use of modifying treatments such as antiplatelet therapy, anticoagulation therapy, administration of fresh frozen plasma, vitamin K therapy, and platelet transfusion. The onset time was determined by emergency records. When onset time was unclear, the case was excluded.

### Detection of leakage sign by image acquisition

**Fig. 1:**
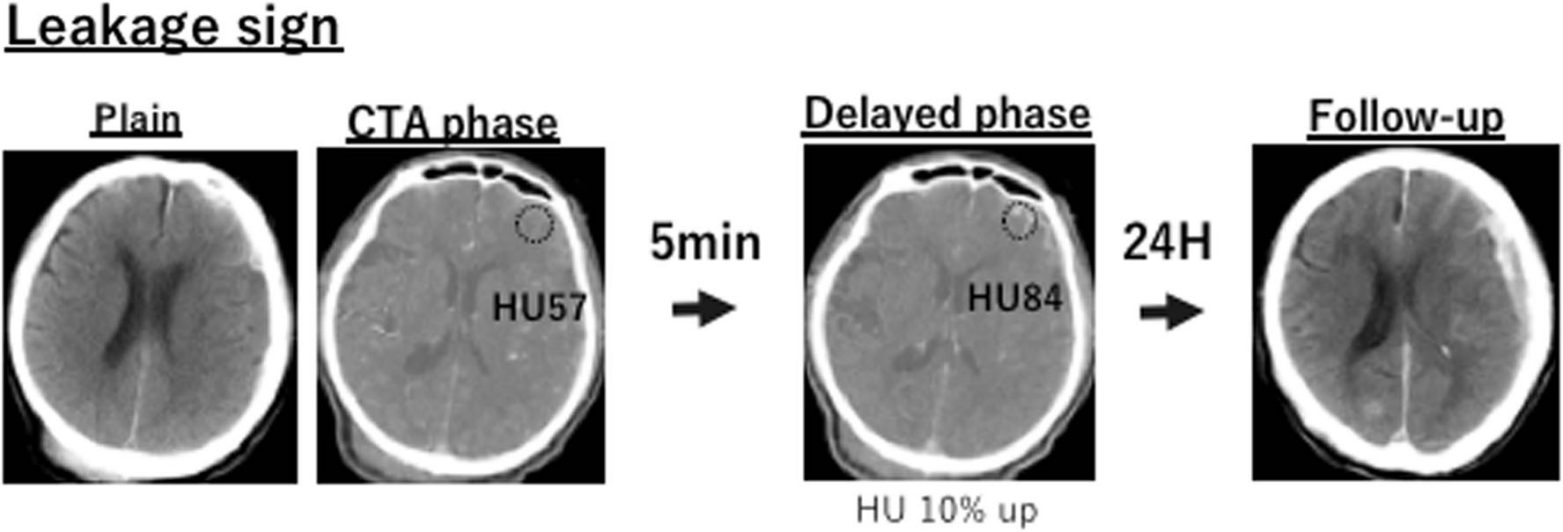
The definition of leakage sign and the clinical examination process used in this study was based on computed tomography angiography (CTA) and delayed phase CT images. The region of interest (ROI; 10-mm diameter) was placed on the delayed phase images to identify leakage of the contrast medium into the hematoma. Hounsfield unit (HU) values in the ROI were determined in each section of the CTA and delayed phase images, and a > 10% increase in HU was considered as a positive leakage sign

The leakage sign method has been previously documented [9, 10]. CT acquisitions were performed according to standard departmental protocols using eight-section General Electric helical CT scanners (BrightSpeed Edge, GE Healthcare, Wisconsin, USA). The first CT scan was performed for CTA (CTA phase), and the second scan (delayed phase CT) was performed 5 min after the CTA (Fig. 1). Plain CT was performed 24 h after the first CT to evaluate the hematoma size and other intracranial findings; a detailed method has been described previously [9].

For the CTA, 70 mL ioversol (Optiray; Fuji Pharma Co., Ltd., Tokyo, Japan; 320 mg I/mL) was intravenously injected at a rate of 3–3.5 mL/s via a power injector through an intravenous line. All plain CT scans were reviewed by two neuroradiologists blinded to the clinical data. The initial and follow-up plain CT studies were evaluated during separate sessions; the images were anonymous and randomized so that the reviewer was blinded to the patient’s identity and the timing of the images (admission or follow-up). The images were evaluated for hematoma size, which was based on the section with the largest hemorrhage size (mm^2^) of all the serial sections.

The following criteria for detection of leakage signs were used: based on the arterial and delayed phase CT images, a region of interest (ROI) of 10-mm diameter was set on the delayed phase images for the leakage of the contrast medium into the hematoma; the HU values in the ROI were determined in each section of the arterial and delayed phase images; a > 10% increase in HU was considered as a positive leakage sign (Fig. 1).

### Measurement of changes in hemorrhage size

A region was set in the selected section that included the region of the hemorrhage, and the area was automatically measured with hemorrhage-HU (60–80) using INFINITT PACS (Infinitt Japan Co.; Japan). The leakage sign is usually present in extra-axial hematomas. To determine if the hematoma size had increased, we compared the measurements of the hemorrhage at initial presentation and at follow-up (24 h later).

### Statistical analysis

Baseline demographics, hematoma volumes, and medication/medical history were compared between leakage sign-positive and leakage sign-negative groups using Fisher exact tests, *t* tests, analysis of variance, or McNemar tests, as appropriate. The relationship between hematoma expansion and leakage sign was analyzed in patients who did not undergo surgery. Statistical analyses were performed using the JMP version 13 software package (SAS Institute Inc., Cary, NC, USA).

## Results

Sixty-seven patients with ASDH (39 men and 28 women) were included in this prospective study. The mean patient age was 72.1 (range, 27–95) years, and the median GCS score at admission was 9 (range, 3–15) points. The leakage sign-positive group had significantly lower GCS scores on admission (*P* < 0.05). There were no significant differences in the distributions of age, sex, platelet count, and international normalized ratio between the leakage sign-positive and leakage sign-negative patients. Contrastingly, the leakage sign-negative group had many patients with a history of hypertension (Table 1).

**Table 1:** Baseline clinical and radiologic characteristics

Characteristic	Total (*N* = 67)		Leakage sign (+) (*N* = 44)		Leakage sign (−) (*N* = 23)		*P* value
	*n* (±SD)	%	*n*	%	*n*	%	
Mean age	72.1 ± 16.3		70.7 ± 17.7		75.3 ± 12.8		0.2379
Sex (male)	39	58.0	28	83	11	48	0.2974
Mean admission blood pressure (mmHg)							
Systolic	148.0 ± 30.0		146.8 ± 33.1		150.5 ± 22.9		0.6437
Diastolic	83.1 ± 19.6		81.9 ± 20.4		85.5 ± 18.1		0.4874
Mean admission GCS	9 ± 4.8		7.7 ± 4.8		11.4 ± 3.9		0.0022*
History of hypertension	27	41.0	11	25	16	72	0.004*
Lab data at admission							
Mean admission platelet count	15.1 ± 5.2		14.6 ± 5.3		16.2 ± 5.0		0.2385
Mean admission INR	1.31 ± 0.61		1.33 ± 0.51		1.26 ± 0.77		0.6675
Mean admission aPTT	33.6 ± 15.2		34.2 ± 2.3		32.4 ± 3.1		0.6375
Altered coagulation	9	13.4	6	13.6	3	13	1
Antiplatelet therapy	10	14.9	6	13.6	4	17.4	0.7263

*P < 0.05

**Fig. 2:**
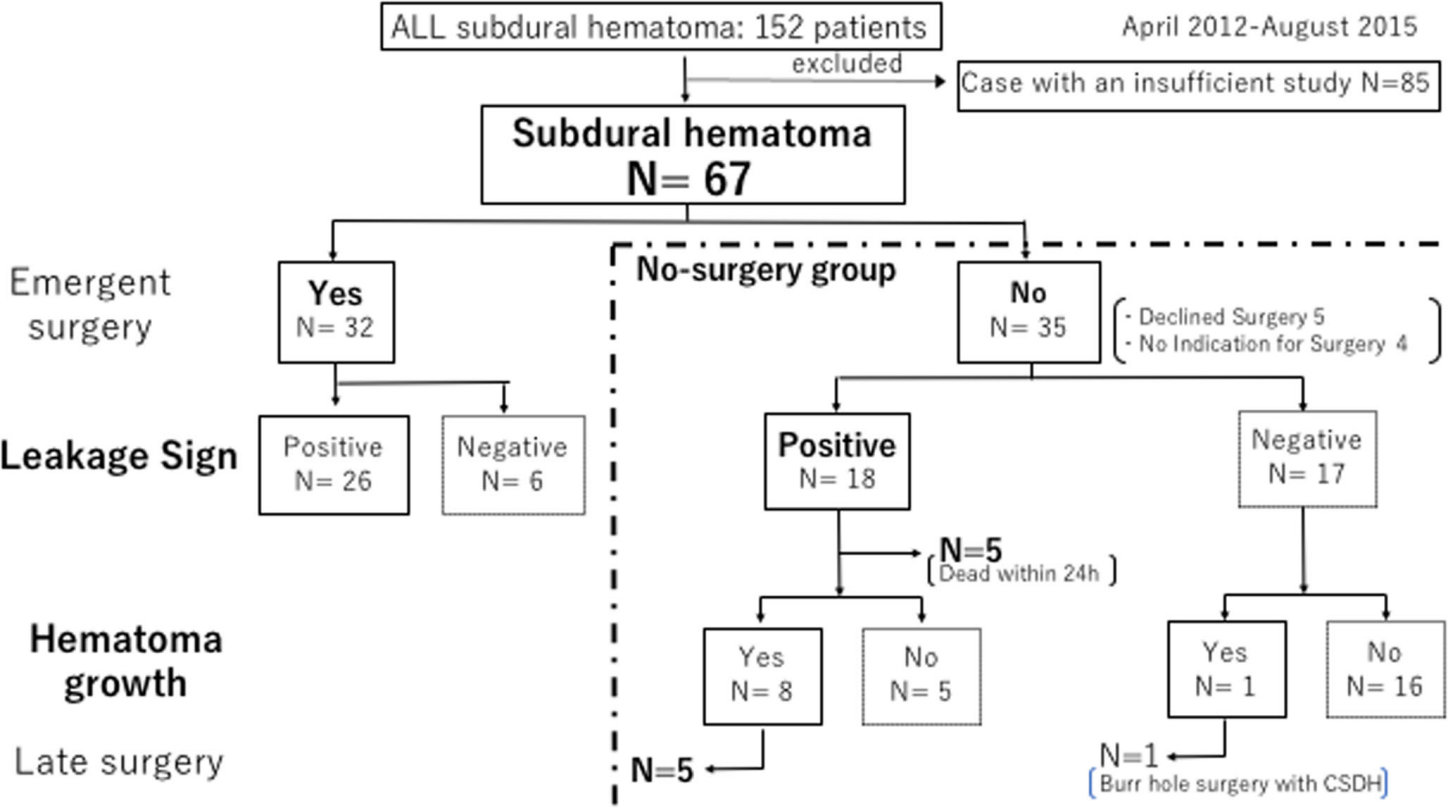
Patient flow in this study

The leakage sign was positive in 44 patients (65.6%) (Table 1). The clinical course of all ASDH patients is shown in Fig. 2.

Emergent hematoma evacuation at admission was performed in 32 patients (47.7%); 26 of these 32 patients (81.2%) were leakage sign-positive. Of the remaining 35 patients, 5 were treated by delayed hematoma evacuation because they showed a decrease in consciousness level or late hematoma expansion; all 5 cases were leakage sign-positive. In the no-surgery group (*n* = 35), 17 patients were leakage sign-negative; one patient with subacute subdural hematoma experienced hematoma expansion. The other 18 patients in the no-surgery group were leakage sign-positive; 8 leakage sign-positive patients (44.4%) experienced hematoma expansion, of which 5 patients died within 24 h. Among all patients, 38 (56.7%) experienced poor outcomes (severe disability or death), including 22 patients (32.8%) who died during hospitalization.

### Relationship between hematoma expansion and predictive value of the leakage sign

**Fig. 3:**
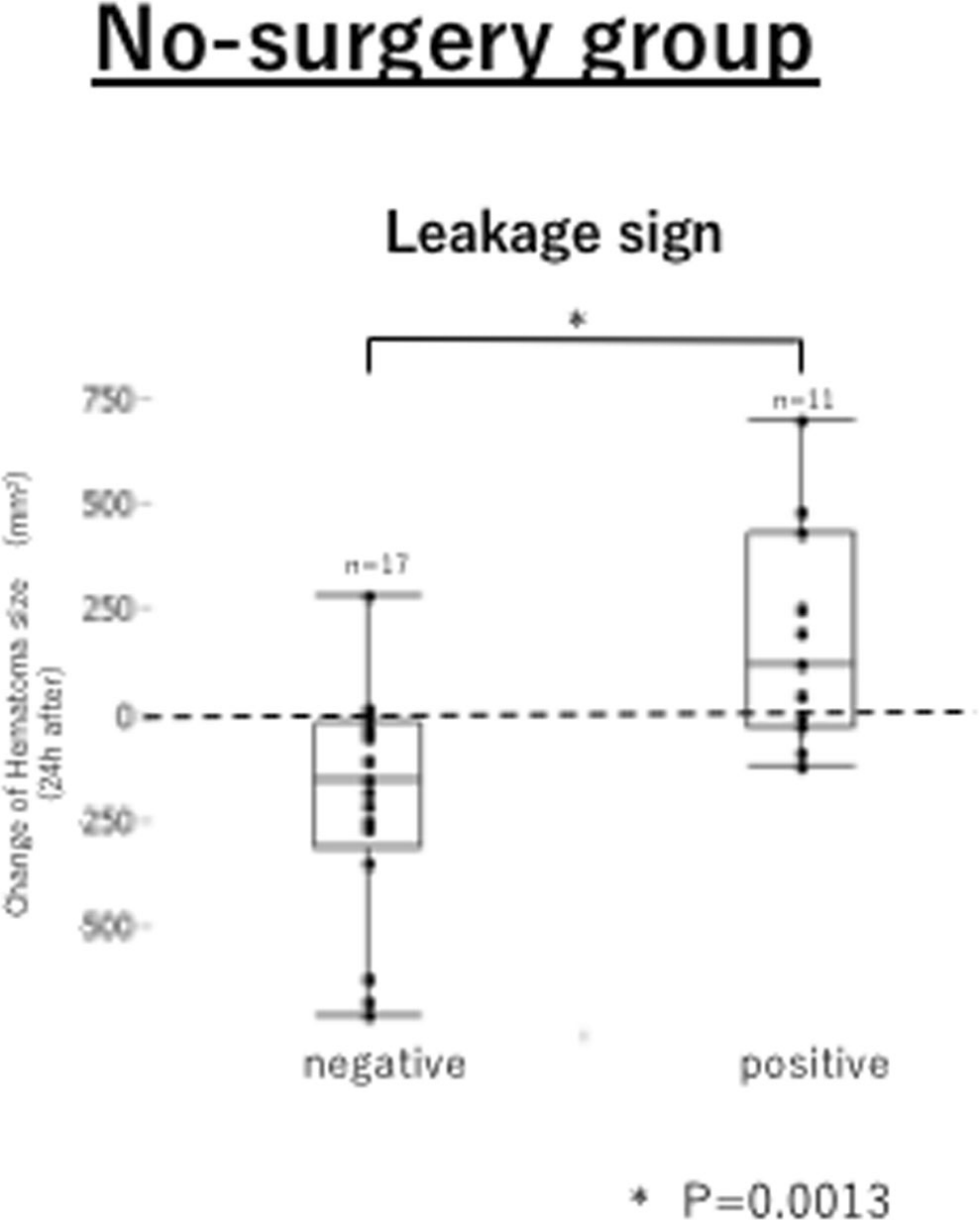
Relationship between change in hematoma size and leakage sign. Change in hematoma size during the 24-h period after admission, as assessed using imaging studies for leakage signs

The relationship between hematoma expansion and leakage sign was analyzed in 35 patients who did not undergo emergent surgery at admission. Of these, 18 patients were leakage sign-positive; the 5 patients who died within 24 h were excluded from the analysis. Nine of the remaining 30 patients experienced hematoma expansion, and 8 of these 9 patients (88.8%) were leakage sign-positive (Fig. 2). The leakage sign showed high specificity (88.8%) and sensitivity (76.1%) for hematoma expansion (Table 2). Patients with a positive leakage sign showed a significantly greater increase in maximum hematoma size than patients with negative leakage signs (182.1 ± 263.9 mm^2^ vs − 198.1 ± 268.9 mm^2^; *P* < 0.05) (Fig. 3). Patients with negative leakage signs showed a decrease in hematoma size 24 h after imaging.

**Table 2:** No-surgery group

Leakage sign		LS (+)	LS (−)	Total	Sensitivity
Hematoma expansion	(+)	8	1	9	88.8%
	(−)	5	16	21	Specificity
	Total	13	17	30	76.1%

**Fig. 4:**
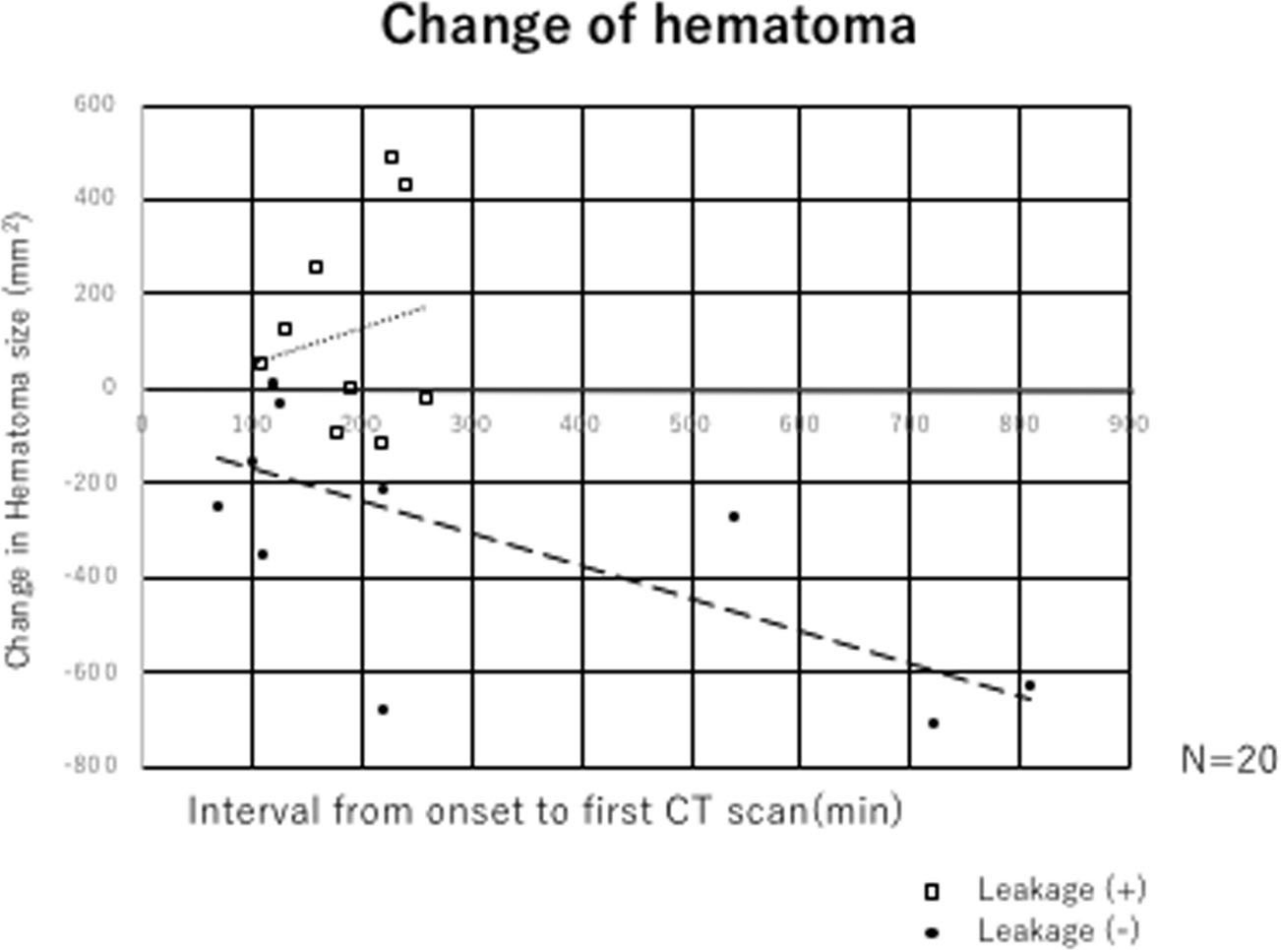
Relationship between change in hematoma size and leakage sign. Dot blot analysis, with the *x*-axis indicating change in hematoma size 24 h later and the *y*-axis indicating the interval from onset to first CT scan (time in minutes)

We analyzed the relationship between the interval from onset to first CT scan and change in hematoma size after 24 h (Fig. 4). According to our data, positive leakage signs were found until 4.5 h after injury. No cases with positive leakage signs were found after longer time intervals. Most cases (8/11) with negative leakage signs showed a decrease in hematoma size.

All patients who were transferred to our institute more than 5 h after injury were leakage sign-negative and the size of their hematoma had decreased (Fig. 4).

### Leakage sign and clinical outcomes

We analyzed the relationship between outcomes measured by GCS score and the presence of leakage sign. The favorite outcomes (good recovery and moderately disabled on the Glasgow Outcome Scale) were significantly lower in cases with positive leakage signs than in cases with negative leakage signs (34.0% vs 60.8%; positive vs negative; *P* < 0.05). In the surgical group, the favorite outcomes were significantly lower when the leakage sign was positive than when it was negative (34.6% vs 66.6%; *P* < 0.05).

## Discussion

Our prospective study of ASDH showed that the presence of leakage signs is closely related to hematoma growth and poor outcomes. The leakage sign-positive group was ranked as severe according to the GCS score on admission (Table 1). Previous reports have shown that in leakage sign-positive cases, hematoma expansion occurs in intracerebral hemorrhage [9] and contusional hematoma [10]. Many previous studies have attempted to develop methods for the prediction of hematoma expansion in patients with intracerebral hemorrhage. Specific signs such as the blend sign and black hole sign have been used to predict the expansion of hematomas in a cerebral hemorrhage without using contrast media [7, 8, 15, 16]. However, there have been few reports that focused on traumatic hemorrhagic diseases. Furthermore, among all methods that use predictive signs observed in brain scans, detection of leakage signs has the highest sensitivity and specificity. Contrast media is frequently used in trauma cases for whole body scans to detect other possible hemorrhagic lesions, and the leakage sign could be an important predictor in traumatic patients.

The detection of spot signs is capable of revealing the extravasation of contrast media on CTA and predicting patient prognosis [1–4, 12, 13], but few studies have examined predictive factors in patients with acute subdural hematomas.

Our results indicated that the presence or absence of leakage signs can predict hematoma expansion within 24 h of scanning with high sensitivity (88.8%) and specificity (76.1%) (Table 2). Furthermore, our study showed that in leakage sign-negative cases, acute subdural hematomas tend to decrease in size (Fig. 3), and that these decreases are more pronounced with longer time intervals between injury and CT scanning. This phenomenon was not observed in leakage sign-positive cases. We think that the hematoma may be washed away by cerebrospinal fluid, once the bleeding stops. In stark contrast, the hematoma size generally increased in cases with positive leakage signs (Fig. 4). Thus, with passing time, hematomas may be more likely to decrease in size in the absence of a leakage sign.

**Fig. 5:**
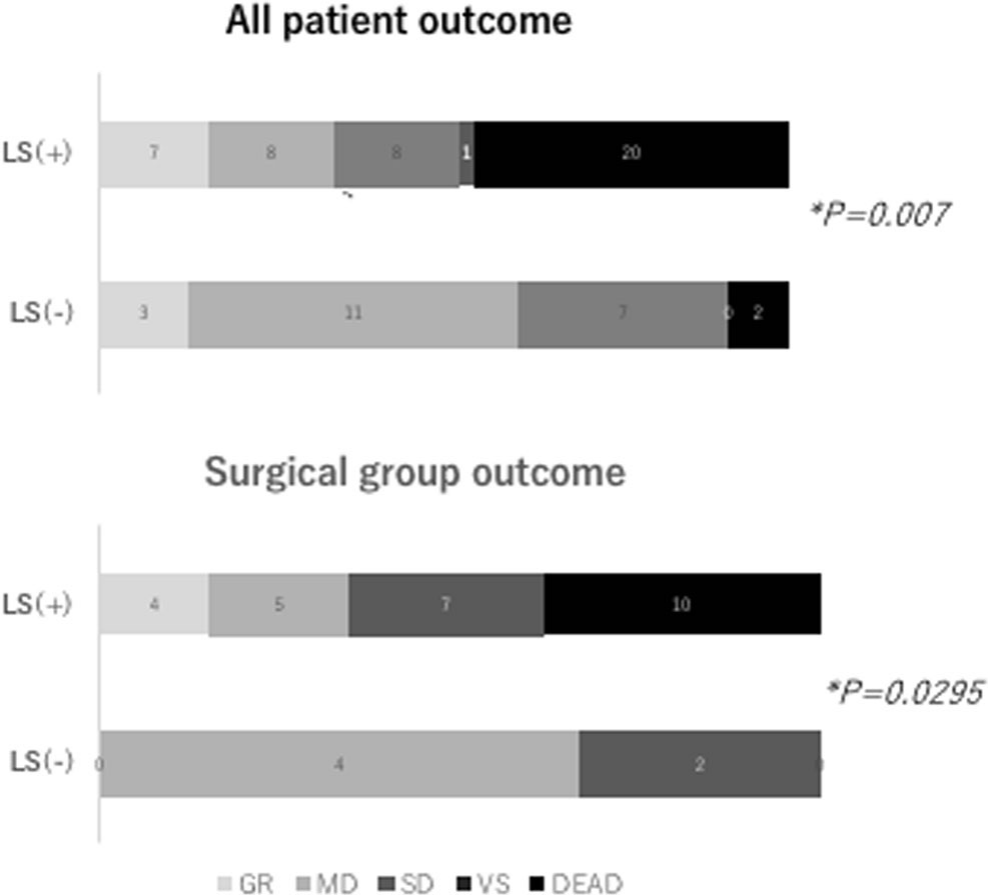
Association between outcome and leakage sign

The leakage sign cannot predict clinical outcomes in patients with contusional hematomas directly [10]. However, the presence of a leakage sign on CT of patients with ASDH was found to be significantly associated with poor outcomes. Patients who received emergent evacuation of hematoma on admission showed the same trend (Fig. 5). This finding indicated that ASDH affects the prognosis more strongly than brain contusion. Therefore, early identification of this sign and aggressive management with rapid surgical evacuation could be very important, even if the patient’s neurological condition does not appear serious.

It may be critical to even wait for 5 min to perform a CT scan. In the present study, we excluded patients exhibiting anisocoria, unstable vital signs, or sudden deterioration in consciousness level, although no serious complications were observed during CT examination. However, measurement of the vital signs and observations that are in a state are necessary when I consider the possibility that a state turns worse. I may exceed a risk when I think about the possibility that leakage sign can predict the increase of the hematoma. We suggest that 5 min is an appropriate and possibly, a safe time period to delay the second CT and that the clinical data might be more important than the risk.

Thus, detection of leakage signs may be a very useful method in predicting the increase in hematoma size in ASDH as well the patient’s outcome. Selective aggressive treatments for leakage sign-positive patients, such as earlier surgical operation, treatment to decrease excessive blood pressure, and specific hemostat medication [11] may improve outcomes in ASDH patients.

## Conclusions

Leakage signs can be reliably identified and are associated with hematoma expansion and poor outcomes. We expect that this method will be helpful in understanding the dynamics of ASDH in clinical medicine.
